# Annular Pancreas Presenting with Intermittent Duodenal Obstruction in Early Childhood: A Diagnostic Masquerade

**DOI:** 10.3390/reports9010026

**Published:** 2026-01-15

**Authors:** Maria Rogalidou, Georgios Papagiannis, Paraskevi Galina, Evangelia Lykopoulou, Konstantina Dimakou, Alexandra Papadopoulou

**Affiliations:** 1 Division of Gastroenterology & Hepatology, First Department of Paediatrics, ‘Agia Sophia’ Children’s Hospital, National and Kapodistrian University of Athens, 11527 Athens, Greece; 2 First Department of Paediatrics, “Agia Sofia” Children’s Hospital, National and Kapodistrian University of Athens, 11527 Athens, Greece; 3 Radiology Department, “Agia Sofia” Children’s Hospital, 11527 Athens, Greece; 4 Gastroenterology Department, “Agia Sofia” Children’s Hospital, 11527 Athens, Greece

**Keywords:** annular pancreas, persistent vomiting, recurrent emesis, children, duodenal obstruction

## Abstract

**Background and Clinical Significance:** Annular pancreas is a rare congenital anomaly in which pancreatic tissue partially or completely encircles the duodenum, potentially causing duodenal obstruction. Clinical presentation varies from asymptomatic cases to persistent vomiting, feeding intolerance, and failure to thrive, often leading to delayed diagnosis. **Case Presentation:** We report a 2-year and 10-month-old girl with a long-standing history of intermittent, recurrent vomiting since the neonatal period, without growth impairment or other alarming symptoms. Initial imaging suggested proximal duodenal dilation, with suspicion for superior mesenteric artery (SMA) syndrome. Endoscopy revealed mechanical obstruction at the second portion of the duodenum. Contrast-enhanced CT confirmed annular pancreas partially encircling the duodenum. The patient underwent duodeno-duodenostomy with an uneventful postoperative course and complete resolution of symptoms. This case illustrates the diagnostic challenges of annular pancreas in older children with atypical presentations. Multimodal imaging is crucial for accurate diagnosis. Surgical bypass remains the definitive treatment, offering excellent long-term outcomes. **Conclusions:** Persistent or recurrent vomiting in children, even without classic signs such as bilious vomiting or failure to thrive, should prompt consideration of annular pancreas. Early recognition and timely surgical intervention can prevent prolonged morbidity and ensure normal growth and development.

## 1. Introduction and Clinical Significance

Annular pancreas is a rare congenital anomaly characterized by a ring of pancreatic tissue encircling the duodenum, which may cause partial or complete duodenal obstruction. Its embryological basis involves abnormal migration or fusion of the ventral pancreatic bud during development [1,2]. Annular pancreas is typically diagnosed in the neonatal period due to duodenal obstruction. However, presentations in older children and adults have been documented, which can complicate diagnosis [3,4]. The exact prevalence of annular pancreas is difficult to determine because many affected individuals remain asymptomatic throughout life. Evidence from autopsy investigations that included detailed duodenal dissection suggests that this anomaly occurs in approximately 5–15 per 100,000 adults [1,2,3,4].

The clinical spectrum ranges from asymptomatic cases to recurrent vomiting, feeding intolerance, and failure to thrive in pediatric patients [5,6]. The rarity and non-specific presentation frequently contribute to delayed or missed diagnosis, particularly when classic signs such as bilious vomiting are absent [7].

Imaging modalities including abdominal ultrasound, upper gastrointestinal (GI) contrast studies, and computed tomography (CT) play crucial roles in detecting annular pancreas and assessing the degree of duodenal obstruction [8,9]. MRI—including MRCP—represents an equally valuable imaging modality and has the advantage of avoiding ionizing radiation. Recent advances in genetic studies, including exome sequencing, have provided additional insights into the molecular underpinnings of this malformation [10].

Surgical intervention remains the definitive treatment, with various techniques such as duodenoduodenostomy employed depending on the extent of obstruction [11].

This case report presents a toddler with recurrent vomiting since the neonatal period who was ultimately diagnosed with annular pancreas after a diagnostic delay, underscoring the importance of considering this rare congenital anomaly in patients with persistent vomiting, even in the absence of alarming symptoms.

## 2. Case Presentation

A 2-year and 10-month-old girl was referred to our department for evaluation of persistent vomiting. She had a long-standing history of intermittent, recurrent vomiting episodes without a specific pattern or periodicity and no other alarming symptoms. Growth parameters had remained normal throughout this period. Between episodes, she was either asymptomatic or experienced mild symptoms, occasionally vomiting once or twice without affecting her quality of life. A chronological overview of symptoms, investigations, and clinical course prior to admission is summarized in Table 1.

At presentation, the patient was in good general condition, with no additional symptoms or clinical signs. Growth parameters were within the normal range for age (weight, height, and head circumference between the 75th and 90th percentiles). Physical examination was unremarkable. Laboratory evaluation revealed normal hematologic and biochemical results, including inflammatory markers, and no evidence of anaemia or electrolyte disturbances.

Abdominal Radiography demonstrated uneven fluid–air levels bilaterally, a cascade-like gastric appearance, and the absence of the typical gastric air bubble, suggesting proximal intestinal stasis.

**Figure 1:**
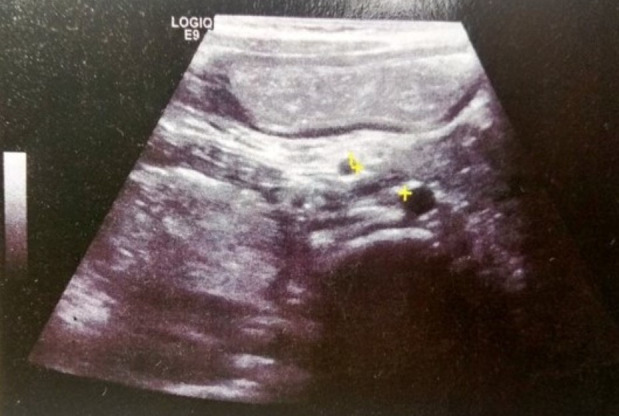
Abdominal Ultrasound proximal duo-denum with a reduced aorto–superior mesenteric artery (SMA) distance of 4 mm.

Abdominal Ultrasound (Figure 1) revealed dilation of the stomach and proximal duodenum with a reduced aorto–superior mesenteric artery (SMA) distance of 4 mm, raising initial suspicion for SMA syndrome. However, mesenteric vessel Doppler ultrasound showed an aorto–SMA angle of 30° (pathological ≤ 25°) and an aorto–SMA distance of 6–10 mm (pathological ≤ 8 mm). These findings were not consistent with SMA syndrome, although duodenal dilation and antiperistalsis persisted, suggesting a mechanical obstruction.

**Figure 2:**
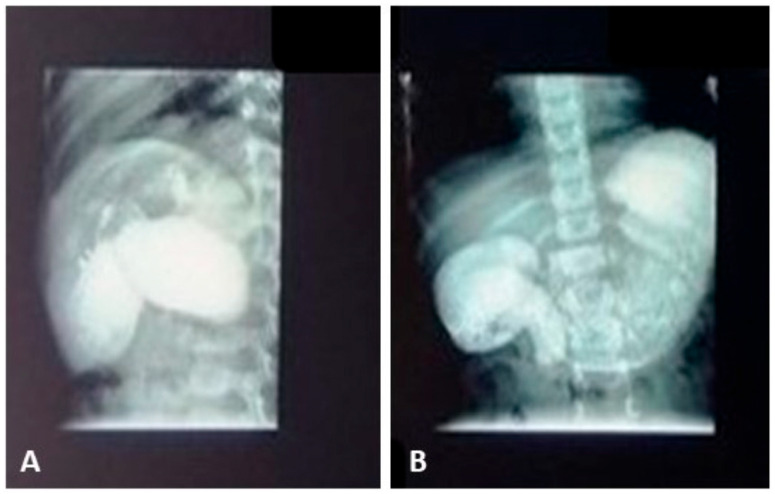
The upper gastrointestinal series demonstrated gastric dilatation with retention of residual food and an incomplete obstruction at the second portion of the duodenum. (**A**) Lateral view showing dilatation of the gastric antrum and the first portion of the duodenum. (**B**) Marked gastric dilatation with incomplete stenosis of the second portion of the duodenum and prestenotic dilatation of the first portion of the duodenum.

Upper Gastrointestinal Series demonstrated a dilated stomach containing residual food and an incomplete obstruction at the second portion of the duodenum, with minimal passage of contrast into the jejunum (Figure 2). At this stage, the differential diagnoses included annular pancreas and incomplete duodenal diaphragm/duodenal web.

**Figure 3:**
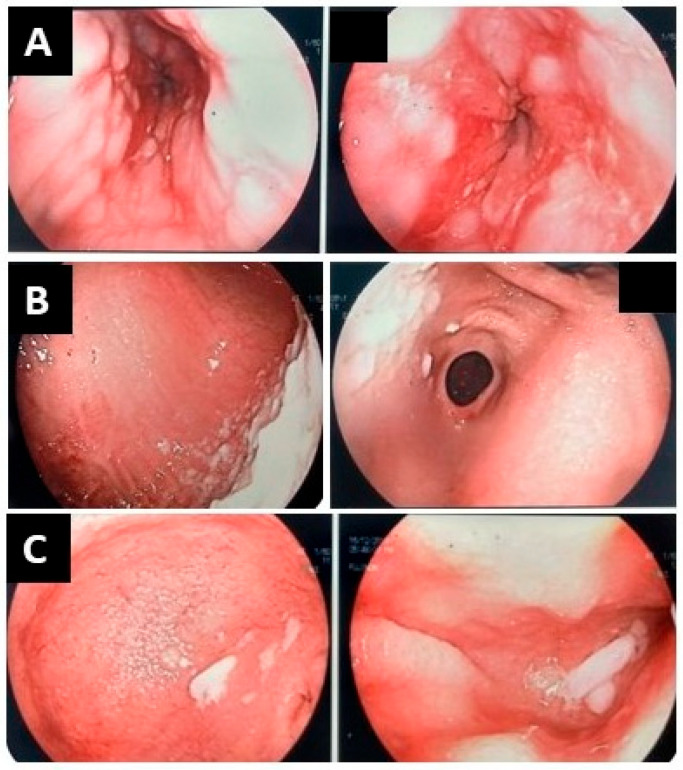
(**A**) Esophagus: Edematous and friable mucosa with ulcerations and residual food. (**B**) Stomach: Mild antral edema and erosions, a patulous pylorus, and retained food in the body and fundus. (**C**) Duodenum: Edematous mucosa in the bulb and second–third portions with food residues; the scope could not be advanced beyond the second portion due to obstruction.

Endoscopic Evaluation revealed esophagitis, gastritis, and duodenitis with retained food debris throughout the upper GI tract. Between the first and second portions of the duodenum, the endoscope could not be advanced further, supporting the suspicion of a mechanical obstruction (Figure 3).

**Figure 4:**
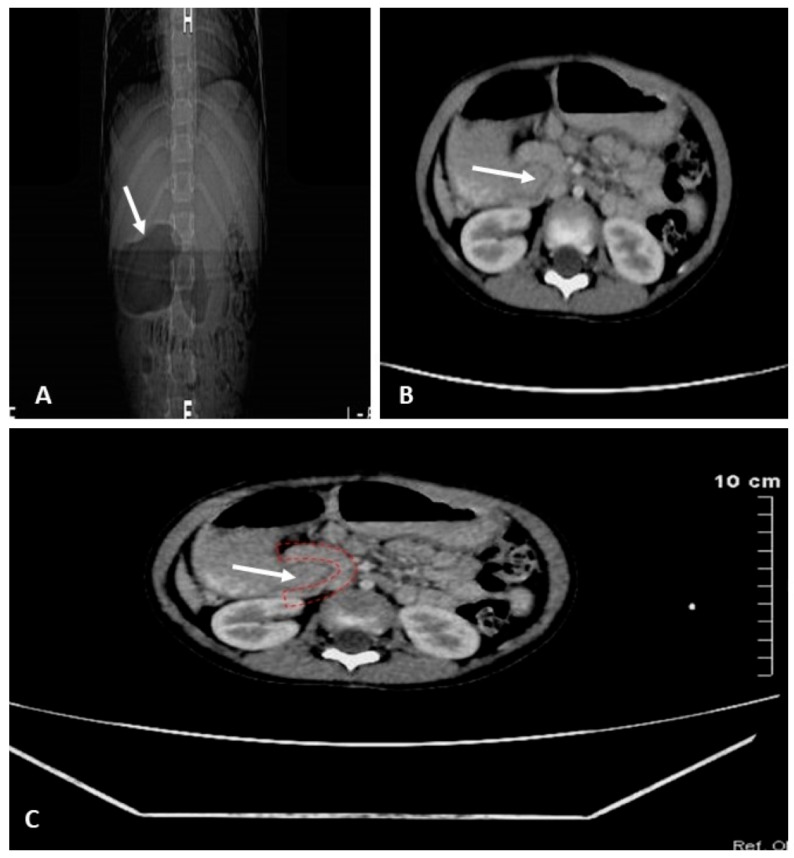
Abdominal CT: Cross-sectional imaging demonstrated pancreatic tissue partially encircling the second portion of the duodenum (arrows), associated with marked dilation of the stomach and proximal duodenum. (**A**): CT Scanogram: Dilatation of the duodenum and gastric antrum. (**B**,**C**): Annular pancreas (red) causing extrinsic compression of the duodenum with resulting proximal (prestenotic) dilatation (arrows).

Contrast-enhanced CT of the abdomen demonstrated pancreatic tissue partially encircling the second portion of the duodenum, associated with marked dilation of the stomach and proximal duodenum. These findings were consistent with annular pancreas, confirming the diagnosis of partial duodenal obstruction secondary to annular pancreatic tissue (Figure 4).

### Management and Outcome

Following diagnosis, the patient underwent a duodeno-duodenostomy. The postoperative course was uneventful, with gradual reintroduction of oral feeding and complete resolution of vomiting. The patient continues to be followed up regularly and remains asymptomatic, with normal growth and development. A repeat endoscopic evaluation one year postoperatively was normal.

## 3. Discussion

Annular pancreas is a rare congenital condition in which pancreatic tissue forms a ring around the duodenum, sometimes causing partial or complete blockage. Annular pancreas has been associated with maternal polyhydramnios and congenital abnormalities such as Down syndrome, esophageal and duodenal atresia, imperforate anus, and Meckel’s diverticulum. The most common associated conditions with annular pancreas appear in Table 2.

Antenatal suspicion of annular pancreas is uncommon but may arise when prenatal ultrasound detects signs of proximal duodenal obstruction, such as polyhydramnios and the “double-bubble” sign (dilated stomach and proximal duodenum) [12]. These features are more frequently associated with duodenal atresia, but their presence without other structural anomalies should prompt consideration of annular pancreas [12,13]. In our patient, the gestation was normal, with no polyhydramnios, and extended evaluation revealed no other congenital anomalies.

Clinical presentation is highly variable. Our patient exhibited intermittent, non-bilious vomiting since the neonatal period, without failure to thrive or other alarming symptoms. This atypical, mild course aligns with previous reports indicating that annular pancreas may remain subclinical or present subtly, delaying diagnosis [5,6,7]. In older children, recurrent vomiting, postprandial fullness, abdominal distension, or gastroesophageal reflux may mimic functional gastrointestinal disorders or other mechanical obstructions [5,6].

The diagnostic process illustrates the challenges of identifying annular pancreas. Initial imaging with ultrasound and upper gastrointestinal series suggested duodenal dilation but was not definitive. SMA syndrome was considered due to reduced aorto–SMA distance [8]. However, the child’s normal weight, the recurrent rather than persistent vomiting, and the presence of an obstruction at the second portion of the duodenum—rather than the third or fourth portions typically affected in SMA syndrome—made this diagnosis unlikely. Endoscopy revealed retained food debris and mechanical obstruction, but could not precisely locate the anomaly. Ultimately, contrast-enhanced CT identified pancreatic tissue partially encircling the duodenum, confirming the diagnosis. This case highlights the importance of multimodal imaging—ultrasound, upper GI contrast studies, endoscopy, and CT or MRI—in evaluating recurrent emesis and proximal duodenal obstruction [8,9].

Although the molecular basis of annular pancreas has been explored through exome sequencing, routine genetic testing is not yet standard [10]. In a study of 115 infants with annular pancreas, rare heterozygous variants were found in IQGAP1 and NRCAM, which are important for cell movement and organisation, as well as in the known AP-related genes PDX1 and FOXF1. These results suggest that problems with cell migration may disrupt the normal development of the ventral pancreatic bud, contributing to AP, and point to a potential genetic influence in around 15% of cases [10]. Our patient did not undergo genetic analysis; the absence of additional congenital anomalies supports the observation that annular pancreas can occur in isolation [3,4,5].

Surgical management remains the definitive treatment for symptomatic cases, aiming to bypass rather than resect pancreatic tissue. Duodeno-duodenostomy is preferred due to its preservation of physiological continuity, low complication rates, and favorable long-term outcomes [13]. Diamond-shaped anastomotic techniques have been suggested to improve postoperative gastric emptying and reduce stasis [11]. Laparoscopic simple oblique duodenoduodenostomy (LSOD) in children with congenital duodenal obstruction was safe and effective, with no leaks or strictures, early feeding (median 4 days), and a median hospital stay of 7 days. LSOD is a viable minimally invasive option in experienced centres [14]. In a retrospective analysis of 59 neonates with annular pancreas, robotic-assisted duodenoduodenostomy (RAD) was compared with laparoscopic-assisted duodenoduodenostomy (LAD). RAD demonstrated shorter anastomosis time, less blood loss, and earlier drain removal, despite longer total operative time due to docking and the learning curve. Complication rates were similar, and proficiency was achieved after 10–13 cases, suggesting RAD provides superior precision and efficiency once the learning curve is surpassed [15]. Our patient underwent duodeno-duodenostomy with complete symptom resolution and normal growth at follow-up, consistent with outcomes reported in pediatric literature [11,12].

Postoperatively, patients should be monitored for growth, feeding tolerance, and gastrointestinal function, with attention to signs of obstruction or delayed gastric emptying. Follow-up should also consider associated anomalies and any anastomotic complications. Multidisciplinary care with pediatric surgery, gastroenterology, and nutrition teams is recommended to ensure optimal outcomes.

This case emphasises the need for high clinical suspicion for annular pancreas in children with persistent/recurrent vomiting, even when classical features such as bilious vomiting or failure to thrive are absent. Early recognition and appropriate surgical management can prevent prolonged morbidity and ensure normal growth and development.

## 4. Conclusions

Annular pancreas is a rare congenital anomaly that can present with a wide spectrum of clinical severity, ranging from asymptomatic cases to persistent vomiting and duodenal obstruction. This case highlights that even mild, intermittent symptoms since the neonatal period can signify underlying structural pathology. Accurate diagnosis often requires multimodal imaging, including ultrasound, upper GI contrast studies, endoscopy, and CT or MRI. Surgical bypass, such as duodenoduodenostomy, remains the definitive treatment, providing excellent outcomes with symptom resolution and normal growth. Clinicians should maintain a high index of suspicion for annular pancreas in children with unexplained, recurrent vomiting, even in the absence of classic features like bilious vomiting or failure to thrive.

## Figures and Tables

**Table 1:** Chronological Overview of Symptoms, Investigations, and Clinical Course.

Age	Clinical Features/Symptoms	Investigations/Findings	Notes/Interpretation
**Neonatal period**	Feeding-related vomiting	—	Partially improved with anti-regurgitation formula
**3.5 months**	Vomiting, afebrile urinary tract infection	Voiding cystourethrogram: VUR grade II–III right, grade I left	Hospitalized; supportive care
**10 months**	Persistent vomiting	Abdominal and cranial ultrasound: normal	No structural abnormality detected
**17 months**	Nocturnal vomiting, poor appetite, abdominal distension, foul-smelling eructations, vomitus containing old food	—	Suggestive of delayed gastric emptying
**20 months**	Vomiting with diarrhea	Abdominal US: fluid-filled intestinal loops, hyperperistalsis	Celiac disease testing negative
**2.5 years**	Frequent vomiting (3–4/day)	Labs: ammonia, lactate, amylase, blood gases, immunologic profile, celiac antibodies all normal; Sweat chloride and skin allergy tests negative	No metabolic or allergic cause identified

**Table 2:** Associated anomalies with annular pancreas.

Associated Anomalies with Annular Pancreas [4,6]
Down syndrome
Malrotation (intestinal malrotation)
Intestinal fixation anomalies
Tracheoesophageal fistula
Esophageal atresia
Duodenal atresia
Imperforate anus (anal atresia)
Meckel’s diverticulum
Congenital heart defects

## Data Availability

The original contributions presented in this study are included in the article. Further inquiries can be directed to the corresponding author.

## Abbreviations

The following abbreviations are used in this manuscript:

SMA	Superior mesenteric artery
GI	Gastrointestinal
CT	Computed Tomography
U/S	Ultrasound
